# Validation of a modified clinical risk score to predict cancer-specific survival for stage II colon cancer

**DOI:** 10.1002/cam4.352

**Published:** 2014-12-08

**Authors:** Raymond Oliphant, Paul G Horgan, David S Morrison, Donald C McMillan, In collaboration with the West of Scotland Colorectal Cancer Managed Clinical Network

**Affiliations:** 1 University Department of Surgery, Faculty of Medicine, University of Glasgow, Glasgow Royal InfirmaryGlasgow, G4 0SF, U.K; 2 West of Scotland Cancer Surveillance Unit, Institute of Health and Wellbeing, University of GlasgowGlasgow, G12 8RZ, U.K; 3 West of Scotland Colorectal Cancer Managed Clinical Network, Glasgow Royal InfirmaryGlasgow, G4 0SF, U.K.

**Keywords:** Clinical risk score, colon cancer, Stage II, survival

## Abstract

Many patients with stage II colon cancer will die of their disease despite curative surgery. Therefore, identification of patients at high risk of poor outcome after surgery for stage II colon cancer is desirable. This study aims to validate a clinical risk score to predict cancer-specific survival in patients undergoing surgery for stage II colon cancer. Patients undergoing surgery for stage II colon cancer in 16 hospitals in the West of Scotland between 2001 and 2004 were identified from a prospectively maintained regional clinical audit database. Overall and cancer-specific survival rates up to 5 years were calculated. A total of 871 patients were included. At 5 years, cancer-specific survival was 81.9% and overall survival was 65.6%. On multivariate analysis, age ≥75 years (hazard ratio (HR) 2.11, 95% confidence intervals (CI) 1.57–2.85; *P*<0.001) and emergency presentation (HR 1.97, 95% CI 1.43–2.70; *P*<0.001) were independently associated with cancer-specific survival. Age and mode of presentation HRs were added to form a clinical risk score of 0–2. The cancer-specific survival at 5 years for patients with a cumulative score 0 was 88.7%, 1 was 78.2% and 2 was 65.9%. These results validate a modified simple clinical risk score for patients undergoing surgery for stage II colon cancer. The combination of these two universally documented clinical factors provides a solid foundation for the examination of the impact of additional clinicopathological and treatment factors on overall and cancer-specific survival.

## Introduction

Colon cancer is one of the commonest causes of cancer death in Western Europe and North America.^1^ Despite overall improvements in relative survival over recent decades, many patients still present with locally advanced or metastatic disease.^2^ Even in those undergoing surgical resection with curative intent for stage II colon cancer, between 20 and 30% will die of their disease within 5 years.^2^–^4^ As a result of these poor outcomes there is increasing interest in the use of adjuvant chemotherapeutic regimens in patients with stage II colon cancer who are at high risk of dying from their disease.^5^ With the introduction of nationally coordinated bowel screening programs and the projected rise in the proportion of early stage disease,^6^,^7^ the need to identify those at high risk has become increasingly important.

A previously published clinical risk score^8^ was devised as a starting point for the identification of high-risk stage II colon cancer patients using routinely collected clinical information. The score uses age (<75 or ≥75 years), mode of presentation (elective or emergency), and occurrence of anastomotic leak (yes or no) to simply and reliably aid identification of patients at risk of poorer longer term cancer-specific survival. However, this score used data from an era (1991–1994) prior to the emergence of surgical specialization, advances in preoperative imaging (e.g., routine CT scanning) and in peri- and postoperative patient care.^9^ Validation of this clinical risk score in a newer cohort of patients undergoing surgery for colon cancer is therefore required.

Therefore, the aim of this study was to evaluate and externally validate a clinical risk score to predict 1-, 3-, and 5-year cancer-specific survival in patients undergoing surgery for stage II colon cancer.

## Methods

Clinical audit data of patients undergoing surgery for AJCC stage II (TMN pT3-4 pN0 pM0) colon cancer in 16 hospital sites from 1 January 2001 to 31 December 2004 were extracted from the prospectively maintained database of the West of Scotland Colorectal Cancer Managed Clinical Network. Individual patient records were then linked to the Scottish Cancer Registry (SMR06). Details included age, gender, deprivation (DEPCAT), site of tumor, mode of presentation, speciality of surgeon, anastomotic leak, and adjuvant therapy.

Colon cancers (C18) were classified according to their anatomical site as per the International Classification of Disease version 10 (ICD-10). Tumors of the rectosigmoid (C19), rectum (C20), anus and anal canal (C21), and appendix (C18.1) were excluded. Mode of presentation was defined as emergency if presentation was with significant rectal bleeding, intestinal obstruction, perforation, or other presentations resulting in an unplanned emergency hospital admission. All other routes of presentation were considered elective. The extent of spread was assessed by conventional AJCC staging classification based on histologic examination of the resected specimen. Individual surgeons were identified as colorectal specialists or nonspecialists by panel members of the corresponding local multidisciplinary team (MDT) of the surgical units under study using a similar method as previously described.^10^ These assessments were made without prior knowledge of the outcome and before any analyses were performed. Anastomotic leak was defined as a composite of both radiological and clinical leaks diagnosed <30-days of surgery. Patients' socioeconomic circumstances were inferred using the 2001 DEPCAT, a validated categorical score that ranks residential postcodes from 1 (most affluent) to 7 (most deprived) using four Census variables that were found to best predict health outcomes—car ownership, unemployment, overcrowding, and lower occupational social class (IV and V).^11^ They were further grouped into three conventional categories: 1 and 2 (affluent); 3–5 (intermediate); and 6 and 7 (deprived).

Patient records were linked to the General Registry Office for Scotland (GROS) death records. Survival time was calculated from date of surgery to date of death or censor with a minimum of 5-year follow up (date of censor 31 December 2010). Postoperative mortality was defined as any death occurring within 30 days of initial surgery. Those dying within this period were excluded from further analyses. Cancer-specific deaths were determined as a first, or principal underlying cause of death with International Classification of Disease (ICD) 10 codes for colorectal cancer, C18 to C20, as well as C26 and C80. Overall survival was determined as death from any cause.

Grouping of the variables was carried out using conventional categories. Univariate and multivariate survival analysis and calculation of hazard ratios (HRs) were carried out using Cox's proportional hazards model. The method used to calculate the original score has been published previously 8 and was used to validate the score in the present cohort. Cumulative survival following surgery was estimated using the Kaplan–Meier method and the log-rank used to test for independence between variables. Predictive model analysis using receiver operating characteristic analysis was carried out. C-statistics were calculated with the null hypothesis that the true area under the curve was 0.5, and asymptotic 95% confidence intervals (95% CIs) calculated around the best estimate. *P* < 0.050 was considered statistically significant throughout. Analysis was performed using the Stata software package version 11 IC (Statacorp, College Station, TX).

The West of Scotland Cancer Surveillance Unit obtained permission to obtain cancer registry data both from Caldicott Guardians of all health boards in the West of Scotland and from the Information Services Division of the NHS in Scotland privacy advisory committee. Permission to obtain clinical audit data was granted by the West of Scotland Colorectal Cancer Managed Clinical Network advisory board.

## Results

Of the 937 patients who underwent a resection for stage II colon cancer, there were 66 (7.0%) postoperative deaths and 871 patients were included in the analysis. The majority were aged <75 years (61.4%), were not socioeconomically deprived (72.7%), presented electively (76.0%) and were treated by a specialist colorectal surgeon (66.6%). A total of 16 (1.8%) patients developed an anastomotic leak and 111 (12.7%) received adjuvant chemotherapy. At 5 years, cancer-specific survival was 81.9% and overall survival was 65.6%.

The relationship between clinicopathological characteristics and cancer-specific survival is shown in Table1. On univariate analysis, age (*P* < 0.001), specialty of surgeon (*P* = 0.038) and mode of presentation (*P* < 0.001) were significantly associated with cancer-specific survival. On multivariate analysis of these factors, age ≥75 years (HR 2.11, 95% CI = 1.57–2.85, *P* < 0.001) and emergency presentation (HR 1.97, 95% CI = 1.43–2.70, *P* < 0.001) were independently associated with cancer-specific survival (Table1).

**Table 1:** Relationship between clinicopathological characteristics and cancer-specific survival in patients undergoing surgery for stage II colon cancer: univariate and multivariate analysis

Risk factor	Patients (*n* = 871)	Univariate HR (95% CI)	*P*-value	Multivariate HR (95% CI)	*P*-value
Age in years (<75/≥75)	535/336	2.08 (1.55, 2.81)	<0.001	2.11 (1.57, 2.85)	<0.001
Gender (Male/Female)	426/445	0.81 (0.60, 1.09)	0.159		
Deprivation^1^ (intermediate) (deprived)	134/499/238	1.10 (0.70, 1.71)	0.684		
		1.30 (0.76, 1.99)	0.410		
Mode of presentation (elective/emergency)	662/209	2.01 (1.48, 2.74)	<0.001	1.97 (1.43, 2.70)	<0.001
Specialization (yes/no)	580/291	1.38 (1.02, 1.87)	0.038	1.17 (0.85, 1.60)	0.333
Leak (no/yes)	855/16	1.01 (0.32, 3.16)	0.987		
Chemo (no/yes)	760/111	1.06 (0.70, 1.60)	0.794		

1 Baseline—affluent.

The relationship between clinicopathological characteristics and overall survival is shown in Table2. On univariate analysis, age (*P* < 0.001), mode of presentation (*P* < 0.001), and adjuvant chemotherapy (*P* = 0.003) were significantly associated with overall survival. On multivariate analysis of these significant factors, age ≥75 years (HR 2.64, 95% CI = 2.64–3.3, *P* < 0.001) and emergency presentation (HR 1.70, 95% CI = 1.36–2.14, *P* < 0.001) were independently associated with overall survival (Table2).

**Table 2:** Relationship between clinicopathological characteristics and overall survival in patients undergoing surgery for stage II colon cancer: univariate and multivariate analysis

Risk factor	Patients (*n* = 871)	Univariate HR (95% CI)	*P*-value	Multivariate HR (95% CI)	*P*-value
Age in years (<75/≥75)	535/336	2.69 (2.18, 3.33)	<0.001	2.64 (2.1, 3.3)	<0.001
Gender (Male/Female)	426/445	0.86 (0.69, 1.06)	0.146		
Deprivation^1^ (intermediate) (deprived)	134/499/238	1.19 (0.86, 1.66)	0.288		
		1.35 (0.95, 1.92)	0.095		
Mode of presentation (elective/emergency)	662/209	1.63 (1.30, 2.05)	<0.001	1.70 (1.36, 2.14)	<0.001
Specialization (yes/no)	580/291	1.21 (0.98, 1.51)	0.081		
Leak (no/yes)	855/16	1.00 (0.44, 2.24)	0.994		
Chemo (no/yes)	760/111	0.56 (0.39, 0.82)	0.003	0.83 (0.56, 1.23)	0.352

1 Baseline—affluent.

**Figure 1:**
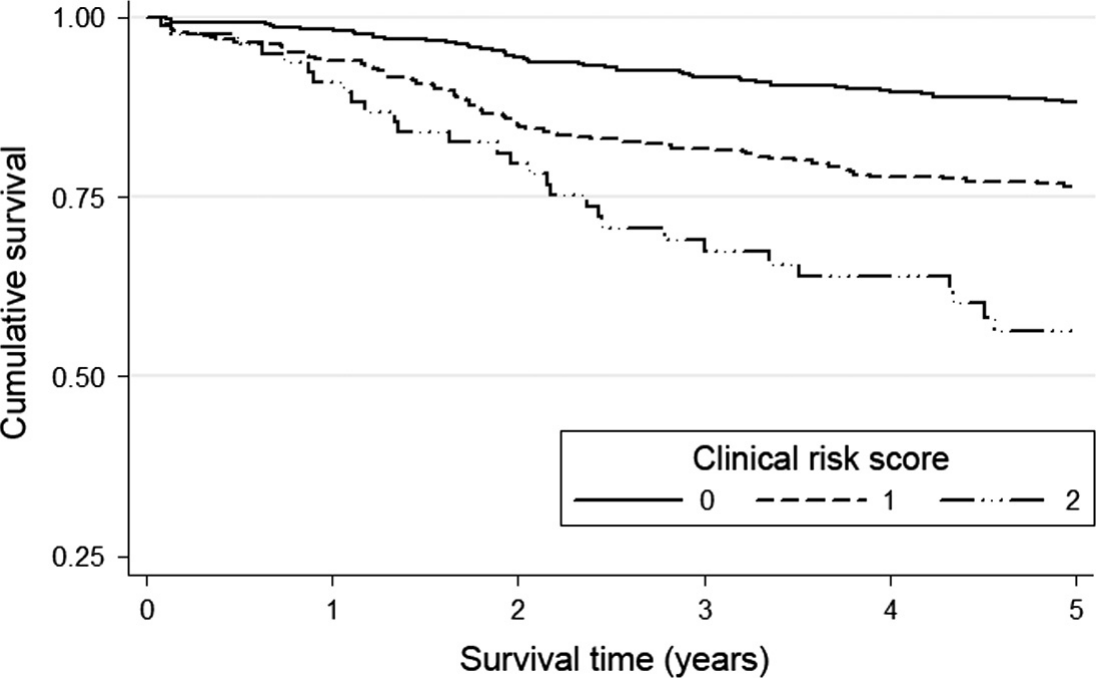
The relationship between clinical risk score and cancer-specific survival in patients undergoing surgery for stage II colon cancer (Log-rank *P* < 0.001).

With reference to cancer-specific survival in colon cancer, as the magnitude of the covariates (HRs) of age ≥75 years (2.11) and emergency presentation (1.97) were similar, they could be allocated a score of 1 if they occurred and 0 if absent. Together these factors could be simply added to form a modified clinical risk score (excluding anastomotic leak) from 0 to 2. The relationship between such a cumulative prognostic score and 1-, 3-, and 5-year cancer-specific survival is shown in Figure1.

In patients with stage II colon cancer, the cancer-specific survival rates at 3 years for patients with a cumulative score 0 was 91.9%, 1 was 82.7%, and 2 was 74.4% (Table3). The cancer-specific survival rates at 5 years for patients with a cumulative score 0 was 88.7%, 1 was 78.2%, and 2 was 65.9%. The area under the curve for the clinical risk score with cancer-specific mortality as an end point at 1, 3, and 5 years was (0.658, 95% CI = 0.582–0.733, *P* < 0.001), (0.629, 95% CI = 0.581–0.678, *P* < 0.001), and (0.626, 95% CI = 0.582–0.670, *P* < 0.001), respectively.

**Table 3:** The relationship between a clinical risk score and cancer-specific survival in patients undergoing surgery for stage II colon cancer

				Cancer-specific survival (%)		
	Stage II n=871 (%)	HR (95% CI)	*P*-value	1-year	3-year	5-year
Clinical score 0	408 (46.8)	1.00		98.3	91.9	88.7
Clinical score 1	381 (43.7)	2.19 (1.53, 3.14)	<0.001	94.0	82.7	78.2
Clinical score 2	82 (9.40)	4.24 (2.65, 6.79)	<0.001	92.7	74.4	65.9

## Discussion

This study shows that in a large cohort of patients undergoing surgical resection for stage II colon cancer, a simple clinical risk score using age and mode of presentation clearly identifies differences in 1-, 3-, and 5-year cancer-specific survival. Unlike the previously published version of this score,^8^ anastomotic leak was not associated with cancer survival in this series and was therefore not used a covariable in the modified score construct. Despite this modification, this simple clinical risk score identified variations in 5-year cancer-specific survival of between 88.7% and 65.9% in those with stage II colon cancer.

Contrary to our findings, a number of large cohort studies indicate that postoperative anastomotic leakage is associated with poor long-term survival in colorectal and gastric cancer.^12^–^14^ The apparent lack of association in our study may be partially explained by the relatively low anastomotic leak rate (1.8%) and the total number of patients included was not large enough to detect a significant survival disadvantage. In addition, those experiencing a leak are more likely to have died within 30-days of surgery and as such excluded from this analysis. The diagnosis of anastomotic leakage may also have changed since the time period from which the original clinical risk score was described (1991–1994) due to the increased use cross-sectional imaging (e.g., CT scanning). Previously, anastomotic leakage would have been diagnosed during an unplanned return to theater in the immediate postoperative period or by using less sensitive contrast radiology techniques. Thus, better imaging techniques are likely to lead to an increase in the number of leaks detected. However, over this period the proportion of anastomotic leakage diagnosed after colorectal cancer surgery in the West of Scotland fell from 4.4% to 2.3% (*P* = 0.002).^2^ This suggests that patient selection and surgical techniques have improved leading to fewer anastomotic leaks. In addition, the management of those diagnosed with anastomotic dehiscence is likely to have changed with an increase in nonoperative strategies to treat those with smaller leaks.^15^ In addition, the proportion of patients treated by a specialist colorectal surgeon increased from 13.8% to 67.6% (*P* < 0.001).^2^ This increase in specialist surgery contributed significantly to improvements in longer term survival and the overall reduction in anastomotic leakage over this period. However, specialty of surgeon was not independently associated with outcome in this study.

The influence of older age (≥75 years) and emergency presentation as predictors of poor outcome after colon cancer surgery have been previously described.^16^–^18^ The negative influence of advanced age on cancer-specific outcomes could relate to increased levels of comorbidity, frailty, and chronic systemic inflammation among the elderly.^18^,^19^ These factors in turn not only influence the type of treatment received by elderly patients, but may also lead to an augmented inflammatory response which is associated with poorer cancer-specific survival.^20^ Other factors such as differences in tumor site and mode of presentation also influence cancer-specific outcomes in the elderly.^21^

Emergency presentation of stage II colon cancer with blood loss, perforation, or obstruction was associated with poorer cancer-specific survival in this study. Those presenting as an emergency were therefore more likely to have had abnormal physiology and attenuated inflammatory response at time of surgery leading to poorer short and longer term cancer-specific outcomes.^16^,^17^,^20^

There is now a large body of evidence that selected patients with stage II colon cancer can derive a small but significant survival benefit from adjuvant chemotherapy.^5^ During the period of this study, it was not routine clinical practice for patients with stage II colon cancer to be considered for adjuvant therapy out with the confines of a clinical trial. The finding that increased age and emergency presentation were associated with poorer outcomes in this study suggests that such patients may derive benefit from adjuvant chemotherapy if fit to receive such treatment. However, the current evidence base in these groups is small. Therefore, further clinical trials of adjuvant chemotherapy in these high-risk groups are required.

This modified clinical risk score has important implications. First, this score can be used as simple and reliable way for clinicians to identify those patients at high risk of developing recurrence and dying of their cancer. Second, these scores provide simple stratification factors for clinical studies and trials. Third, the score may provide a basis for future staging systems for stage II colon cancer to which recognized tumor prognostic factors such as intra or extramural vascular invasion, peritoneal involvement, margin involvement, tumor perforation, tumor grade, number of examined lymph nodes, inflammation-based scores (e.g., modified/optimized Glasgow Prognostic Score), and comorbidity might be added.^22^–^25^ The identification of high-risk patients who would benefit from adjuvant chemotherapy is therefore crucial to improve outcomes. However, no widely adopted reliable method of predicting response to adjuvant chemotherapy for stage II colon cancer has been developed or incorporated into routine clinical practice. The identification of reliable clinicopathological and robust molecular prognostic markers to enable stratified individual patient-specific treatment is therefore highly desirable.

In summary, the results of this study validate and modify the use of a simple clinical risk score for patients undergoing surgery for stage II colon cancer. The score provides a solid foundation for the future examination of the impact of additional clinicopathological and treatment factors on prediction of cancer-specific survival in stage II colon cancer with the clinical implication of identifying high-risk patients who may benefit from adjuvant therapies.
